# Magnetic Nanomaterials as Contrast Agents for MRI

**DOI:** 10.3390/ma13112586

**Published:** 2020-06-05

**Authors:** Sofia Caspani, Ricardo Magalhães, João Pedro Araújo, Célia Tavares Sousa

**Affiliations:** IFIMUP and Departamento de Física e Astronomia, Faculdade de Ciências Universidade do Porto, Rua do Campo Alegre 687, 4169-007 Porto, Portugal; sofixsofi@gmail.com (S.C.); ricardo.magalhaes@fc.up.pt (R.M.); jearaujo@fc.up.pt (J.P.A.)

**Keywords:** nanomaterials, Gd-based contrast agents, Mn-based contrast agents, iron oxide nanoparticles, magnetic nanodiscs, synthetic antiferromagnetic nanostructures, nanowires, contrast agents, MRI, theragnosis

## Abstract

Magnetic Resonance Imaging (MRI) is a powerful, noninvasive and nondestructive technique, capable of providing three-dimensional (3D) images of living organisms. The use of magnetic contrast agents has allowed clinical researchers and analysts to significantly increase the sensitivity and specificity of MRI, since these agents change the intrinsic properties of the tissues within a living organism, increasing the information present in the images. Advances in nanotechnology and materials science, as well as the research of new magnetic effects, have been the driving forces that are propelling forward the use of magnetic nanostructures as promising alternatives to commercial contrast agents used in MRI. This review discusses the principles associated with the use of contrast agents in MRI, as well as the most recent reports focused on nanostructured contrast agents. The potential applications of gadolinium- (Gd) and manganese- (Mn) based nanomaterials and iron oxide nanoparticles in this imaging technique are discussed as well, from their magnetic behavior to the commonly used materials and nanoarchitectures. Additionally, recent efforts to develop new types of contrast agents based on synthetic antiferromagnetic and high aspect ratio nanostructures are also addressed. Furthermore, the application of these materials in theragnosis, either as contrast agents and controlled drug release systems, contrast agents and thermal therapy materials or contrast agents and radiosensitizers, is also presented.

## 1. Introduction

Magnetic Resonance Imaging (MRI) is one of the most powerful techniques in medical imaging, due to its noninvasiveness and radiation-free nature. Compared with other clinical imaging techniques, MRI presents several advantages associated with image flexibility and high spatial resolution, good contrast in soft tissues and also the ability to provide information related to blood circulation and blood vessels. On the other hand, its major disadvantage is the low sensitivity [1]. However, in recent decades, several types of contrast agents have been developed to improve the MRI sensitivity and enhance the information present in the images, namely by using magnetic ions and magnetic nanoparticles (NPs) [2].

Contrast agents based on Gadolinium(III), so called Gd(III)-based (GBCAs), are among the most widely used contrast agents in MRI. About 40% of MRI scans are performed with GBCAs, and, in the case of neuro MRI exams, GBCAs are used about 60% of the time [3]. However, GBCAs have raised various toxicity concerns associated with systemic fibrosis, which is a potentially fatal condition.

Also, a certain percentage of the administrated GBCAs can persist in the organism for long periods, usually in form of Gd(III) [4]. Superparamagnetic iron oxide nanoparticles (SPIONs) have been developed and approved as possible substitutes for GBCAs. Such particles have distinct characteristics, namely, biocompatibility, easy metabolization, high saturation magnetic moments that can be controlled by tuning the particle size and composition, and easy surface functionalization [5]. However, these contrast agents were not commercially successful [3]. This may be attributed to the fact that they are size-restricted by the superparamagnetic regime, which limits the magnetic moment of each particle, and, through simulations, it was verified that the optimal dimensions of particles for MRI contrast agents surpass such superparamagnetic threshold [6]. Among the various nanomaterials that can be found in the literature with different shapes and compositions, magnetic nanostructures (MNS), and in particular, nanodiscs and nanowires, are promising alternatives to SPIONs due to their larger magnetic moments that are not restrained by the superparamagnetic regime [7]. Also, MNS are a promising system for theragnostics, since they can be used as contrast agents and, at the same time, generate localized heating inside the body with the use of an external alternating current (AC) magnetic field, or be used for controlled drug delivery, photodynamic therapy and neutron capture therapy [8,9].

This review is focused on the recent advances in magnetic nanoparticles as contrast enhancing agents in MRI. Consequently, first we will discuss the principles of MRI regarding the use of T_1_ and T_2_ contrast agents, addressing, simultaneously, the contrast agents most widely used in clinical practice. Then, we explore recent reports regarding the development of new types of contrast agents based on MNS: SPIONs, nanodiscs, synthetic antiferromagnets and high aspect ratio nanowires. Furthermore, the use of these nanostructures in both cancer diagnosis and therapeutics will also be discussed.

## 2. T_1_ and T_2_ Contrast Agents

Contrast agents are used to improve the MRI images of tissues and organs by changing the relaxation times of the water protons and, therefore, modifying the magnitude of the signal in the body regions where the agent is incorporated [10]. An MRI contrast agent normally shortens the rates of all of the relaxation processes; however, each substance predominantly influences one of them. Contrast agents that mostly shorten the relaxation time of the longitudinal component of the magnetization are called T_1_, or positive contrast agents, while the T_2_, or negative, contrast agents, mainly reduce the relaxation time of the transverse component [2]. Usually, two main parameters are used to evaluate the performance of a contrast agent: longitudinal relaxivity (r_1_) and relaxivity ratio, i.e., transversal relaxivity (r_2_)/longitudinal relaxivity (r_1_). Here, the value of r_1_ is related to the signal enhancement potential of a contrast agent, while the r_2_/r_1_ ratio is used to define the category within which a contrast agent fits, i.e., either positive (T_1_) or negative (T_2_). Generally, contrast agents that have a low r_2_/r_1_ ratio (<5) are classified as T_1_ substances, while agents that have a larger r_2_/r_1_ ratio (>10) are categorized as T_2_ contrast media [11].

### 2.1. T_1_ Contrast Agents

**Figure 1:**
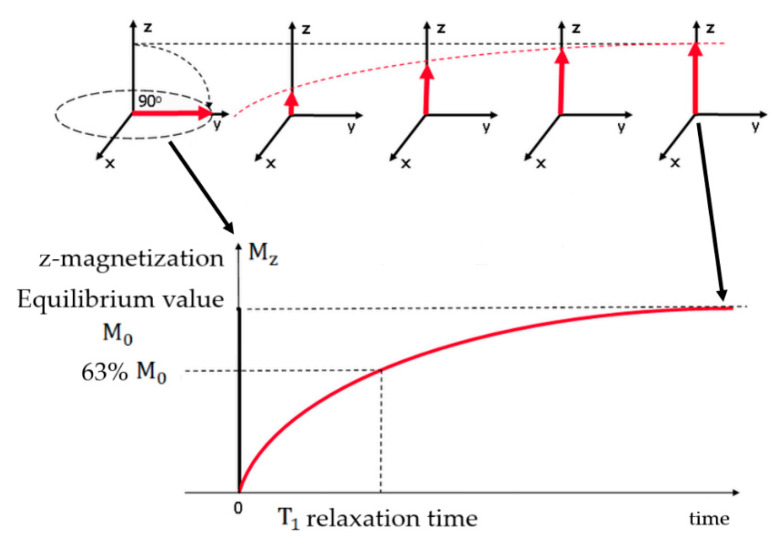
T_1_ relaxation process. Diagram of the T_1_ relaxation when a 90° RF pulse was applied to a system at equilibrium. M_z_ is reduced to zero, but then returns gradually to its equilibrium value if no further RF pulses are applied. Adapted from Ridgway, J.P. “Cardiovascular magnetic resonance physics for 7clinicians: Part I.” J. Cardiovasc. Magn. Reson. 12 (2010) 71.

The longitudinal relaxation is directly related to the energy loss from the spin to its neighbors and represents the realignment process of the longitudinal component of the magnetization with the external magnetic field. When a strong external magnetic field (B_0_) is applied to a living tissue, the magnetic moments of the hydrogen nuclei, which are naturally randomly oriented, will be oriented parallel or antiparallel to the field. The energy difference between these two states is very small and originates a net magnetization vector (M_z_) that does not produce any measurable signal due to its static equilibrium state. To obtain information from the spins, a radiofrequency (RF) pulse at the Larmor frequency, i.e., the frequency at which the nuclei freely precess about B_0_, must be applied. Through this interaction, it becomes possible to identify two relaxation processes resulting from the application of a pulse that causes M_z_ to flip 90° from the positive z-axis to the transverse one. After the RF transmitter is switched off, each individual magnetic moment will begin to precess about B_0_ at its own Larmor frequency, and an equilibrium state will be sought. This means that the transversal magnetization will decay over time (free induction decay (FID)) due to the dephasing of the magnetic moments, originating a signal that oscillates at the Larmor frequency, and the longitudinal component of the magnetization will return to its initial maximum value along the direction of B_0_ [12]. In this context, the T_1_ relaxation time provides a measure of how fast the net magnetization vector returns to its initial state parallel to B_0_. This parameter is defined as the time required for M_z_ to recover to approximately 63% of its equilibrium value after the application of an RF pulse, as represented in Figure 1 [13]. Consequently, the MRI image can be improved by reducing the T_1_ relaxation time, which yields a bright contrast in the acquired pictures. This can be achieved by using positive contrast agents, such as paramagnetic ions or materials.

Complexes of gadolinium (Gd(III)), manganese (Mn(II)) and iron (Fe (III)) are widely adopted as paramagnetic T_1_ contrast agents in MRI. Gd(III) is characterized by seven unpaired electrons in the valence 4f orbital, while Mn(II) and Fe(III) contains five unpaired electrons in the d subshell. Nevertheless, all of them present high magnetic moments, large longitudinal electronic relaxation times (∼10^−8^ s) and no magnetization in the absence of an external magnetic field [3,10,14,15]. There are several transition and lanthanide metals with unpaired electrons, but to be an efficient contrast agent, the electron spin-relaxation time in the metal needs to match the Larmor frequency of the protons [2]. Additionally, the main problem associated with paramagnetic heavy metal ions in their native form is their toxicity [16]. Free Gd (Gd(III)), for instance, is very toxic and must be administered in its stable form to prevent the release of the metal ion in vivo. As a result, several types of GBCAs have been developed to satisfy these conditions [3,14]. An octadentate polyaminopolycarboxylato-based ligand is present in Gb-based compounds exhibiting the ninth coordination sites available for water linkage. Among all the possible metal complexes, discrete Gd(III) chelates are the most suitable paramagnetic contrast agents, dominating the field of contrast agents used in clinical practice. Clinically-used GBCAs (as well as the majority of the other contrast agents) can be intravenously or orally administrated, and they are divided into three different groups: blood pool contrast agents (BPCAs), extracellular fluid (ECF) agents and organ-specific agents [10]. ECF agents, due to their low molecular weight, travel first to the heart and are then distributed in both intravascular and cellular media. On the other hand, blood pool agents are considered good contrast agents to image arteries and veins, as, after administration, they are confined to the intravascular space. Organ-specific agents are capable of targeting specific tissues and organs, such as liver, or lymph nodes. The main contrast agents used clinically and some examples that have received approval for clinical trials are summarized in Table 1.

To further reduce the toxicity of the free metal ions and have contrast agents that cross the blood brain barrier (BBB), research in the field of paramagnetic contrast agents has focused, in the last few years, on the development of nanostructured materials [15]. Paramagnetic NPs present several advantages, such as the tunability of size and shape compared to the contrast agents involving free metal ions; therefore, a wide range of approaches have been adopted to produce paramagnetic NPs for MRI. In general, the development of these NPs can be divided in two main classes: nanostructured materials (i.e., Gd_2_O_3_, Mn_3_O_4_, Dy_2_O_3_, MnO) with incorporated paramagnetic ions [19,20,21,22,23]; or the postfunctionalization of the NPs with lanthanide coordination complexes. The latter approach has been developed using different nanoparticle scaffolds as supports (quantum dots, gold, silica and micelles), which allow a subsequent doping to be performed with pentetic acid (DTPA), dodecane tetraacetic acid (DOTA), or derivates [24,25,26,27].

Iron-based NPs are among the most widely used inorganic materials in nanomedicine, and some of them have already been used in clinical trials and practices as MRI contrast agents [28]. In contrast to Gd-based contrast agents, iron oxide NPs are typically negative, i.e., T_2_, contrast agents (e.g., superparamagnetic iron oxide NPs). Since, these types of contrast agents are associated with background interference and low resolution, several studies have been carried out, leading to the development of ultrasmall iron oxide (USIO) NPs [5,28,29,30,31,32,33,34,35] and magnetic nanowires (NWs) [31,36,37,38,39]. The T_1_ enhancement of USIO-NPs and NWs is associated with increased surface area, suppressed magnetization and surface effects on magnetization. Additionally, when the nanoparticles are too small, their magnetization can be easily flipped using thermal energy. Under this condition, their behavior is paramagnetic [40,41,42,43].

Besides the structures mentioned above, other types of structures have been assessed, such as stealth rare earth oxide nanodiscs [44], linear arrays of magnetite nanoparticles [45] and antiferromagnetic compounds [21,46,47].

### 2.2. T_2_ Contrast Agents

**Figure 2:**
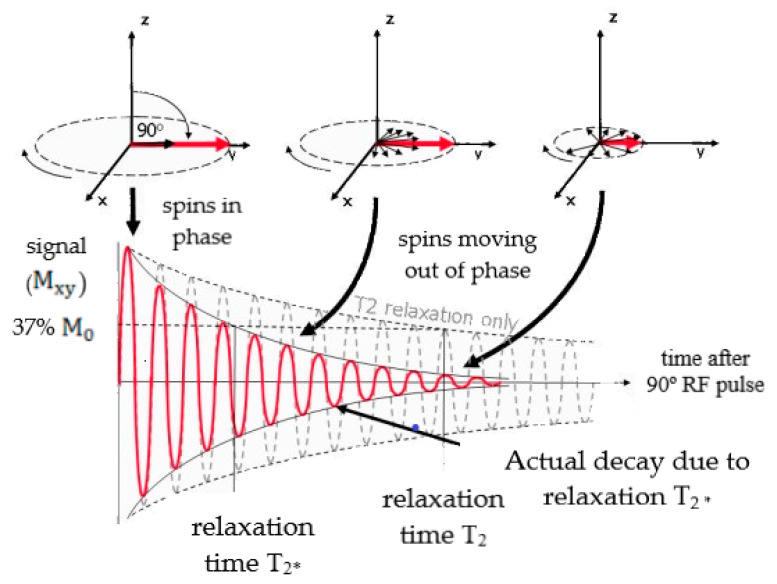
Scheme of T_2_ and T_2_* relaxation when a 90° RF pulse was applied to a system at equilibrium. M_xy_ present a maximum amplitude when the RF pulse is applied, then returns gradually to its equilibrium value. This decaying signal is called Free Induction Decay (FID). Adapted from Ridgway, J.P. “Cardiovascular magnetic resonance physics for clinicians: Part I.” J. Cardiovasc. Magn. Reson. 12 (2010) 71.

Transverse relaxation depends on the spin precession frequency around B_0_, and is defined by the T_2_ relaxation time. This parameter represents the interval of time during which the transverse magnetization decreases to approximately 37% of its initial value, as presented in Figure 2. Firstly, a RF pulse is applied to excite the spins that start to precess in phase. However, the local magnetic environment and spin–spin interactions cause small differences in the Larmor frequency, and the spins start to dephase, leading to T_2_ relaxation, as illustrated in Figure 2 [48]. However, in that same figure, it is possible to verify that the real signal decays faster than the prediction based on the T_2_ relaxation time. In fact, a faster exponential decrease was observed as a function of the T_2_* time constant, which takes into account not only the intrinsic effects associated with T_2_, but also the dephasing resulting from extrinsic magnetic inhomogeneities, such as defects within the main static magnetic field, B_0_, or susceptibility differences between adjacent tissues [49].

Therefore, superparamagnetic iron oxides NPs (SPIONs) have been considered a possible substitute to Gd(III)-complexes due to their high saturation magnetization, biocompatibility, ease of metabolization and surface functionalization [3,10,50,51,52,53,54,55,56,57]. Recently, it was also demonstrated that several types of NPs were able to cross the BBB, increasing the possibility of early diagnosis of several diseases in the brain [58].

The major drawback of these nanoparticles is that the size is restrained by the superparamagnetic regime, which designates the maximum size that a particle can have to sustain zero remanent magnetization, which is a fundamental property, since it prevents the aggregation of particles in the absence of a magnetic field. For this reason, each particle has a constrained magnetic moment, and the optimized particle size for T_2_ MRI contrasts agents (radius of about 20 nm) usually surpasses the superparamagnetic limit [6,59]. To overcome these limitations, several authors have studied various alternatives, namely, high aspect-ratio ferromagnetic NPs [39,60] and synthetic antiferromagnetic (SAF) nanostructures [61].

## 3. Magnetic Properties

The main advantages of NPs are associated with their high surface-to-volume ratios [62,63,64]. Through several studies, it has been stated that the saturation value of magnetization builds up linearly with particle’s dimensions, until it achieves a bulk value. On the other hand, the shape of the particle affects the magnetic properties, and the correlation between magnetization (M) and geometry remains a subject of study for biomedical applications. Regarding the magnetic properties, NPs are usually designed as paramagnetic, diamagnetic, ferromagnetic, ferrimagnetic and antiferromagnetic [62,63].

### 3.1. Paramagnetic Contrast Agents

Paramagnetic contrast compounds involve metal ions/atoms that have uncoupled magnetic moments [10,15,40,62,63]. The unpaired free electrons produce magnetic dipoles which are randomly aligned at equilibrium state, presenting an average magnetic moment equal to zero. Thus, paramagnetic materials have moments with no long-range order, where the dipoles are aligned only when an external magnetic field is applied, and they possess a small positive magnetic susceptibility [62]. Regarding their MRI application, paramagnetic nanomaterials possess many advantages compared to traditional coordination complexes; for instance, their composition, size and shape are readily tunable. The localized density effects enhance the magnetic attributes of nanoparticles, increasing their T_1_ and/or T_2_ relaxivity values when compared to the corresponding coordination complexes. Moreover, longer blood circulation time is facilitated by pharmacokinetics [15].

In addition, it has been stated that paramagnetic properties can also arise from dimensional confinement. In the regime of a single magnetic domain (i.e., superparamagnetism), when the size of the particle is below a critical value, i.e., typically 5 nm [5,30,31,32,33,34,35], the magnetization can be easily flipped by the thermal energy. This leads to a T_1_ contrast improvement that can be attributed to various aspects, such as the suppression of the magnetization, the increased surface iron center exposure, surface effects and water diffusion [40].

### 3.2. Superparamagnetic Nanoparticles

Superparamagnetism in SPIONs arises from paramagnetic iron and is characterized by the zero coercivity and large magnetic moment in the presence of an external magnetic field [50]. SPIONs are single domain nanostructures where a decoupling of the magnetization of the particle from the lattice due to thermal energy overcomes the anisotropy energy [65]. This happens when the sample volume is reduced below a critical value, or superparamagnetic limit, in which more energy is required to create a domain wall than to support the external magnetostatic energy of the single domain state. The magnetic anisotropy energy, E, which is responsible for all the magnetic moments along the same direction, is presented in Equation (1):

(1) $$E\left(\theta \right)=K_{\mathrm{eff}}{\mathrm{Vsen}}^{2}\left(\theta \right)$$

where $K_{\mathrm{eff}}$ is the anisotropy constant, V corresponds to the volume of the nanoparticle and θ is the angle between the easy axis and the magnetization. Therefore, $K_{\mathrm{eff}}$V is the energy barrier between the two reverse easy directions of magnetization that are energetically equivalent. With decreasing particle size, $K_{\mathrm{eff}}$V decreases until the value of thermal energy and fluctuations of the overall magnetic moment can take place within the particle. These thermal fluctuations then affect the orientation of the magnetization if they are large enough to overcome the magnetic anisotropy barrier. This change in magnetization resembles the behavior of a paramagnetic material, albeit involving a magnetic moment that is several orders of magnitude larger, leading to the creation of a superparamagnet [40,51,59]. Two key features describe a superparamagnetic system: lack of hysteresis, and data of different temperatures superimposed onto a universal curve of M vs. H/T [66]. The relaxation time of moment τ is given by the Néel-Brown expression in Equation (2), where k_B_ is the Boltzmann constant, and $\tau_{0}$ ≈ $10^{-9}$ s.

(2) $$\tau =\tau_{0}\exp\left(\frac{K_{\mathrm{eff}}V}{k_{B}T}\right)$$

**Figure 3:**
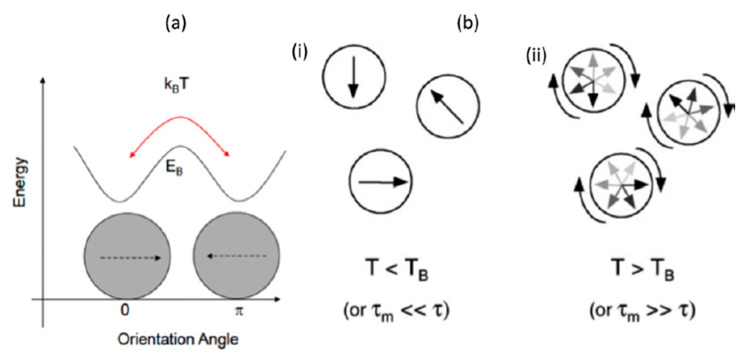
(**a**) Scheme of the energy needed, E_B_, for the magnetization of a nanoparticle to flip between the parallel and antiparallel orientations along the easy axis. (**b**) Superparamagnetic nanoparticles in a (i) quasi-stable blocked state, and (ii) a freely rotating state. Reprinted with permissions from Plouffe, B.; Murthy, S.K.; Lewis, L.H. Fundamentals and application of magnetic particles in cell isolation and enrichment: A review. Rep. Prog. Phys. 78 (2014) 016601. Copyright (2014) Institute of Physics.

If the total magnetization of the particles reverses at shorter times than the experimental timescales, the system will appear to be superparamagnetic. If not, the system is in the blocked state [40,51,59,65,67,68], as presented in Figure 3.

### 3.3. Synthetic Antiferromagnetic

SAFs are a novel type of magnetic nanoparticle; their structure consists of two ferromagnetic layers separated by a nonmagnetic one. The nomenclature of ‘synthetic antiferromagnetic’ refers to the antiparallel alignment of the ferromagnetic layers, which provides almost zero magnetization in the presence of low applied fields [69]. The interaction between two ferromagnetic layers can be either magnetostatic or by interlayer exchange coupling. The former one strongly relies on the aspect ratio of the architecture, while the latter is determined by the choice of the material and the number of atomic layers [70]. Moreover, oscillatory dependence on the thickness of the spacer has been found [71,72]. Furthermore, SAFs are nanostructures which have been optimized to have negligible remanence, low susceptibility around zero field and a distinct, tunable, switch to full magnetization, which allows high saturation magnetization values at low applied fields [61,69,73].

### 3.4. High Aspect Ratio Nanowires

The unusual properties of nanowires (NWs) arise from their high-density of electronic states, high surface to volume ratio and high aspect ratio. In comparison with other low-dimensional systems, NWs have two quantum-confined and one unconfined direction that make it possible to tune their magnetic properties, namely, the Curie temperature, coercivity on both axes, orientation of the magnetic easy axis, saturation field and magnetization and remanence magnetization [7]. Moreover, in NWs with multiple segments along their length, an antiferromagnetic coupling can be induced by controlling the separation between the magnetic layers [74]. Their magnetic properties can be tuned by changing the wire length, diameter, chemical composition and thickness of the segmented layers. NWs can be considered feasible substitutes to spherical NPs, as they interact differently due to their anisotropy properties that arise from the high length to diameter ratio [75,76,77,78]. Moreover, the prevalent shape anisotropy induced by the high aspect ratio and higher magnetic moments make them attractive as contrast agents in MRI, as well as in several other biomedical applications [39].

## 4. Iron Oxide Nanoparticles

NPs are spherical nanostructures with a size between 1 and 100 nm, making them comparable to biomolecules [79,80]. Furthermore, they present unique physical and chemical properties which arise from the fact that a great proportion of their atoms is present on the nanoarchitecture surface [79,81]. These distinct attributes, along with their reduced size, have made these nanoformations a widely studied material in biomedicine, particularly as diagnostic, theranostic or therapeutic tools [81,82]. Nevertheless, only a few elements can be used for such applications due to toxicity problems [80,83]. Within this context, iron oxide NPs have demonstrated great potential, especially as MRI contrast agents, since they possess low toxicity, biodegradability, chemical stability under physiological conditions, and a fast response when an external magnetic field is applied [52,80]. Consequently, various authors have been studying the use of these nanostructures in the context of that medical imaging technique.

**Figure 4:**
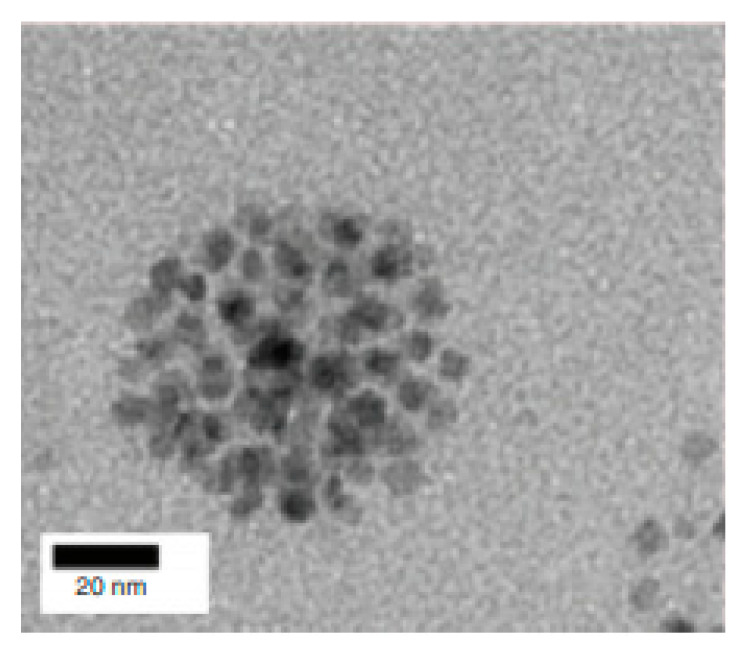
Transmission electron microscopy (TEM) micrograph depicting a raspberry SPION with uranyl acetate (1%) staining. Reprinted from Hobson, N.J.; Weng, X.; Siow, B.; Veiga, C.; Ashford, M.; Thanh, N.T.; Schätzlein, A.G.; Uchegbu, I.F. “Clustering superparamagnetic iron oxide nanoparticles produces organ-targeted high-contrast magnetic resonance images.” Nanomedicine 14 (2019) 1135.

**Figure 5:**
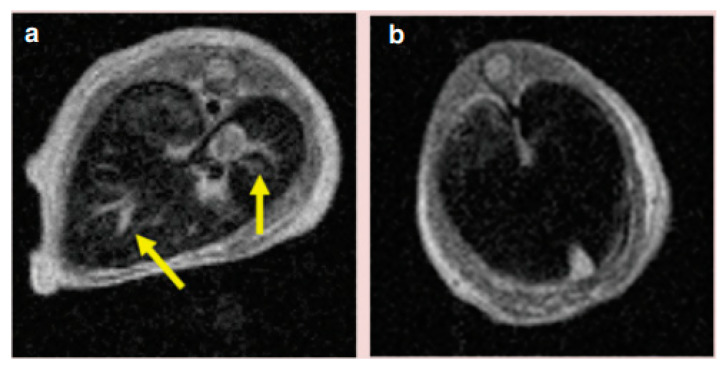
T_2_-weighted axial MRI pictures illustrating a mice liver cross section 60 min postadministration of (**a**) raspberry SPIONs; (**b**) commercially available contrast agent Ferucarbotran®. The arrows in (**a**) show hepatic liver vessels, that are not observed in (**b**). Reprinted from Hobson, N.J.; Weng, X.; Siow, B.; Veiga, C.; Ashford, M.; Thanh, N.T.; Schätzlein, A.G.; Uchegbu, I.F. “Clustering superparamagnetic iron oxide nanoparticles produces organ-targeted high-contrast magnetic resonance images.” Nanomedicine 14 (2019) 1135.

An example is the work from Hobson et al. [84], where the suitability of SPIONs as T_2_ contrast agents was examined. Here, the goal was to improve the contrast produced by NPs in a T_2_-weighted MRI. Therefore, 5 nm spherical nanoarchitectures were fabricated via high temperature thermal decomposition, coated with oleic acid and then agglomerated inside a self-assembling polymer (chitosan amphiphile) through physical means without cross-linking, forming raspberry SPIONs (Figure 4). After the synthesis process, it was verified that these nanostructures were colloidally stable within various biomedical liquids. Afterwards, the MR relaxivities of single, as well as clustered, NPs were measured, as an increase on their spin-spin (r_2_) to spin-lattice (r_1_) relaxation ratio (r_2_/r_1_), i.e., from 3.0 to 79.1 had been noticed when grouping occurred; this gave rise to better negative contrast. Furthermore, the aggregated nanoarchitectures were intravenously administered to mice to analyze their biodistribution and performance in in vivo MRI studies. As a result, it was observed that only the liver and the spleen accumulated the nanostructures, and that these exhibited a blood half-life of 28.3 min. Additionally, MRI tests demonstrated an effective contrast, associated with these raspberry SPIONs, in the two organs where they were accumulated, providing clear images of the liver vasculature, including the portal vein, since they were localized in the extravascular space of that organ (Figure 5).

**Figure 6:**
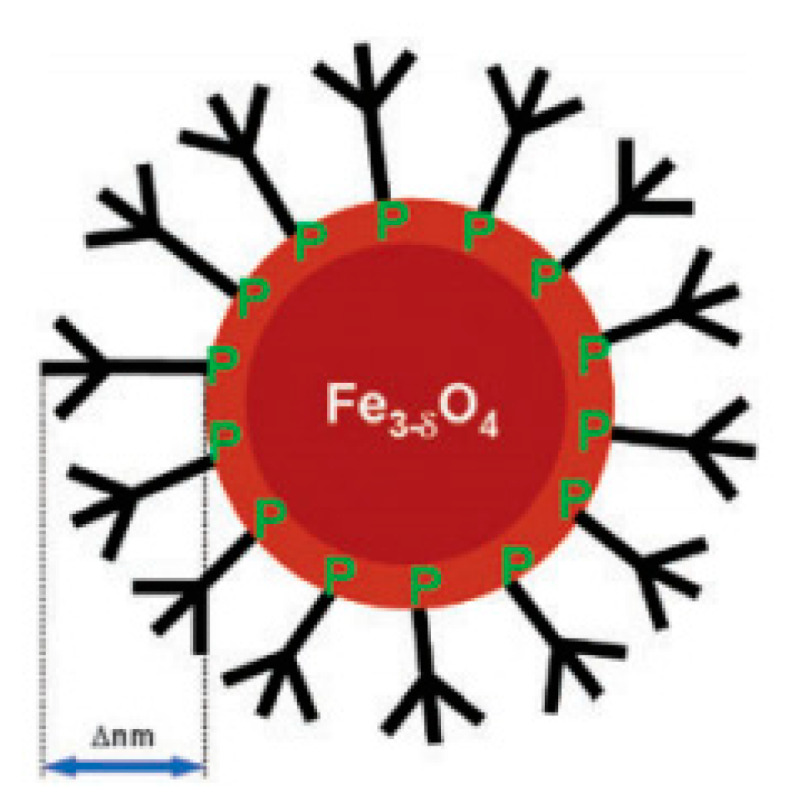
Illustration representing a hydrophilic pegylated dendrons covalently bonded to an iron oxide nanoparticle via a phosphonate anchor. Reproduced with permission from Basly, B.; Felder-Flesch, D.; Perriat, P.; Billotey, C.; Taleb, J.; Pourroy, G.; Bégin-Colin, S. “Dendronized iron oxide nanoparticles as contrast agents for MRI.” Chem. Commun., 46 (2010) 985. Copyright (2010) Royal Society of Chemistry.

Another approach was considered by Basly et al. [85]. Here, the authors covalently bonded hydrophilic pegylated dendrons to SPIONs, using a phosphonate anchor (Figure 6). The dendritic molecules were selected because they were discrete and monodisperse entities, not only exhibiting adjustable characteristics, but also permitting distinct as well as reproducible polyfunctionalizations to be made at their periphery. On the other hand, the phosphonate coupling agent was chosen since it provided a strong binding, stabilized suspension within water at a physiological pH, and conserved the magnetic properties of the nanostructures. Then, the relaxivities associated with these nanoarchitectures were analyzed using a 1.5 T magnetic field. As a result, the authors obtained a r_2_/r_1_ ratio equal to 44.8, having measured relaxivity values 1.5 times higher than those exhibited by commercially available polymer-decorated NPs. Additionally, in vitro relaxivity measurements under 7 T confirmed a significant negative contrast.

**Figure 7:**
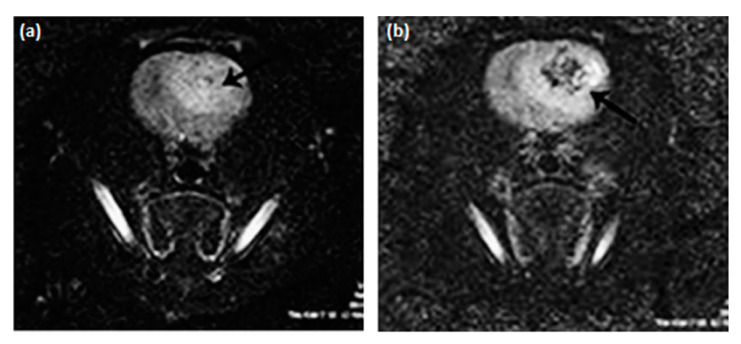
In vivo T_2_-weighted MR pictures representing a rats’ brain with C6 gliomas (indicated by the arrows), acquired 48 h after the administration of (**a**) SPIONs and (**b**) Lf-SPIONs. Reprinted from Xie, H.; Zhu, Y.; Jiang, W.; Zhou, Q.; Yang, H.; Gu, N.; Zhang, Y.; Xu, H.; Xu, H.; Yang, X. “Lactoferrin-conjugated superparamagnetic iron oxide nanoparticles as a specific MRI contrast agent for detection of brain glioma in vivo.” Biomaterials, 32 (2011) 495. Copyright (2010), with permission from Elsevier.

Xie et al. [86] performed a different study in which an MRI contrast agent for the identification of brain gliomas in vivo, i.e., lactoferrin-conjugated SPIONs (LfSPIONs), was developed. After synthesizing NPs, the authors examined their physical, chemical and magnetic properties, as well as their interaction with glioma cells. As a result, a hydrodynamic diameter equal to ∼75 nm, a 51 emu/g Fe saturation magnetization, and a T_2_ relaxivity equal to 75.6 mM^−1^s^−1^ were observed for these spherical nanostructures. Additionally, an in vitro study considering a rat glioma cell line (C6) revealed that the Lf-SPIONs yielded MR images with a better T_2_ contrast than those produced by SPIONs. Furthermore, an in vivo investigation was performed using rat models together with the developed NPs. Considerably improved contrast among the tumor and the neighboring healthy tissues was observed on T_2_-weighted brain glioma MR images up to 48 h after the administration of Lf-SPIONs (Figure 7). Following such a time period, a histochemical analysis led to the observation of those nanostructures around the vascular region of the lesion tissue slices. Real-time polymerase chain reaction (RT-PCR) plus Western Blot were employed in the brain tumor tissues. These techniques allowed the authors to confirm a larger expression level associated with Lf receptors compared to normal tissue from the same organ. Consequently, these results indicated that Lf-SPIONs are adequate T_2_ MRI contrast agents for brain glioma, presenting large selectivity and sensitivity.

**Figure 8:**
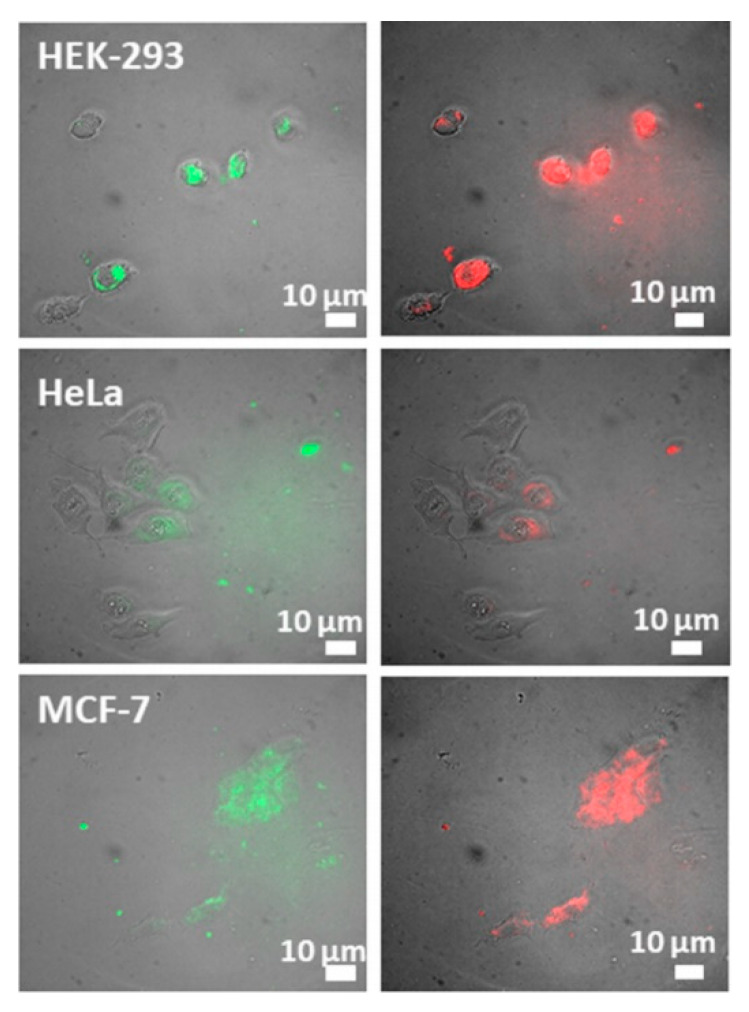
Pictures representing the GO-SPIONs green (550 nm) as well as red (635 nm) fluorescent emissions in healthy (HEK-293) and cancer (HeLa; MCF-7) cells. Adapted from Gonzalez-Rodriguez, R.; Campbell, E.; Naumov, “A. Multifunctional graphene oxide/iron oxide nanoparticles for magnetic targeted drug delivery dual magnetic resonance/fluorescence imaging and cancer sensing.” PLOS ONE 14 (2019) e0217072.

In a different work, Gonzalez-Rodriguez et al. [87] fabricated biocompatible SPIONs conjugated with graphene oxide (GO-SPIONs). These spherical nanostructures presented a mean size of 250 nm and demonstrated the ability to be used in magnetic targeted therapy, fluorescence imaging, cancer discernment through optical pH-sensing, anticancer drug delivery and as MRI contrast agents. Cytotoxicity assays revealed a reduced cell death resulting from the internalization of the nanoparticles at a 15 g/mL concentration. Furthermore, relaxivity measurements indicated a r_2_/r_1_ ratio of ∼10.7 for the GO-SPIONs, being considerably higher than that exhibited by free SPIONs (∼2.3). Consequently, this suggests that graphene oxide-conjugated SPIONs could be employed as T_2_ MRI contrast agents. Additionally, the authors successfully distinguished cancer cells from healthy ones in vitro through the ratios of emission intensity associated with NPs, since they presented fluorescence in the visible range that depended on a medium pH (Figure 8). Concerning drug delivery, the authors were able to perform fluorescence-tracked intracellular delivery of hydrophobic doxorubicin noncovalently conjugated with GO via the application of an external magnetic field. This yielded a 2.5-fold efficacy enhancement compared to the free drug at reduced concentrations, making it possible to reduce the drug dose required to achieve an identical therapeutic effect.

**Figure 9:**
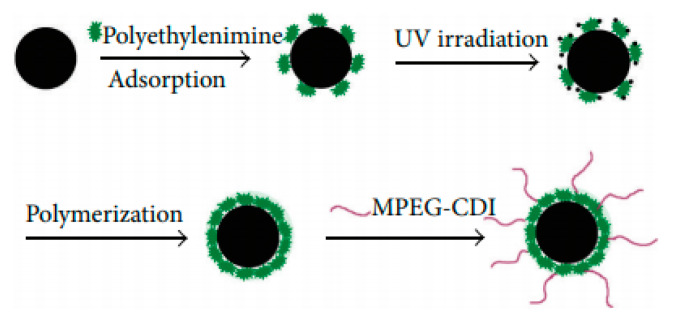
Schematic representation of the MPEG-PEI-SPIONs fabrication process. Reproduced from Zhang, Y.; Zhang, L.; Song, X.; Gu, X.; Sun, H.; Fu, C.; Meng, F. “Synthesis of Superparamagnetic Iron Oxide Nanoparticles Modified with MPEG-PEI via Photochemistry as New MRI Contrast Agent.” J. Nanomater. (2015) 417389.

Also considering SPIONs, Sulek et al. [88] fabricated a contrast agent by the noncovalent functionalization of nanoparticles with peptide amphiphile molecules, which provided water solubility and improved their biocompatibility (Figure 9). Then, after production, the nanocomplexes relaxivity were assessed under a 3.0 T magnetic field; a r_2_/r_1_ ratio as high as 111.55 was observed, i.e., much larger value than for commercially available SPIONs. Furthermore, in vitro incubation experiments using fibroblasts (NIH 3T3) revealed that these functionalized NPs were, in fact, highly biocompatible. Moreover, it was observed that such spherical nanostructures were located on the cell membrane or matrix. Additionally, the hydrophilic peptide sequence located at the SPIONs surface, which supplies stability as well as bioactivity within aqueous conditions, could be changed to target specific tissues.

A different T_2_ contrast agent, i.e., multifunctional polymeric-coated multicore NPs (bioferrofluids), were investigated by Ali et al. [89]. These spherical nanostructures consisted of various maghemite NPs with a hydrophilic polymer (polyethylene glycol, PEG, acrylate). Furthermore, their uptake and toxicity in the liver of mice were assessed through MRI together with histological techniques. Then, the obtained outcomes were compared against those acquired when employing commercially available Endorem magnetic fluids under identical experimental circumstances. The r_2_/r_1_ ratio of the bioferrofluids synthesized by the authors was 184, while that of Endorem was 54.02. Additionally, these NPs not only exhibited a smaller blood circulation period, but were also shown to be efficient reticuloendothelial system agents, since they remained in the liver tissue. It was also observed that those bioferrofluids stayed in the liver for a longer period than Endorem. Nevertheless, no perceptible histological lesions in the examined liver were caused by the two contrast agents analyzed over a period of 60 days postadministration.

**Figure 10:**
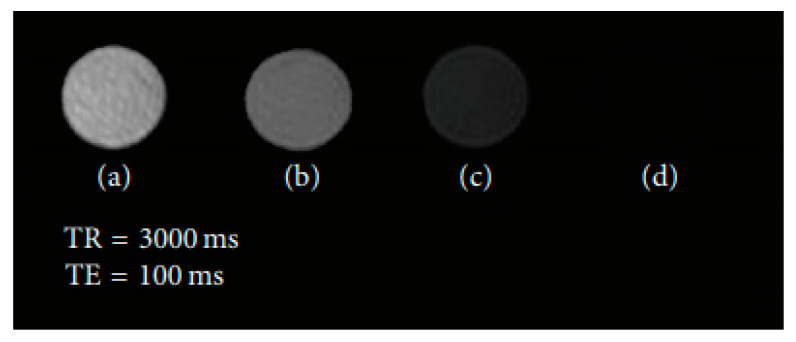
T_2_-weighted MR pictures representing MPEG-PEI-SPIONs at various concentrations, namely (**a**) 0.063; (**b**) 0.125; (**c**) 0.250; (**d**) as well as 0.500 mg/mL. Reproduced from Zhang, Y.; Zhang, L.; Song, X.; Gu, X.; Sun, H.; Fu, C.; Meng, F. “Synthesis of Superparamagnetic Iron Oxide Nanoparticles Modified with MPEG-PEI via Photochemistry as New MRI Contrast Agent.” J. Nanomater. (2015) 417389.

Another type of contrast agent was analyzed in a work from Zhang et al. [90]. The nanomaterials consisted of SPIONs coated with polyethylenimine (PEI), which were obtained through photochemistry, and whose superficial layer was altered by poly(ethylene glycol) methyl ether (MPEG), MPEG-PEI-SPIONs (Figure 9). The physical properties, stability and MRI feasibility of these NPs were assessed. They were shown to possess a hydrodynamic size of 34 nm. Their coating was determined by a Fourier transform infrared spectrometer to be 31% and 12% proportions of PEI and MPEG, respectively. Additionally, magnetic measurements showed superparamagnetic behavior, as well as 46 emu/g saturation magnetization. Furthermore, a stability test indicated that MPEG-PEI considerably enhanced the spherical nanostructure stability. Relaxation measurements demonstrated similar r_2_ values for both PEI-SPIONs as well as MPEG-PEI-SPIONs. Additionally, T_2_-weighted MR images using MPEG-PEI-SPIONs revealed a considerable improvement of the MR signal, as the concentration of those NPs in water increased (Figure 10). This indicated that these spherical nanostructures are able to produce large magnetic field gradients near their surface.

Yue-Jian et al. [91] studied a novel contrast agent consisting in antifouling PEG-coated SPIONs. Monodisperse oleic acid-coated SPIONs were synthesized via the thermolysis of iron oleate, followed by the self-assembling of spherical nanostructures and the PEG-lipid conjugates in water. Then, it was observed, through dynamic light scattering, that the PEG-coated SPIONs were stable within water at pHs from 3 to 10 and at sodium chloride concentrations up until 0.3 M. Their incubation with a cell culture medium possessing 10% fetal bovine serum, which replicated the in vivo plasma, confirmed such stability, with no changes in the NP dimensions after 24 h having been noted. These results indicated an absence of protein adsorption upon the surface. In vitro relaxation measurements indicated a greater r_2_ for these spherical nanoarchitectures than that associated with the commercially available contrast agent, Feridex IV.

**Figure 11:**
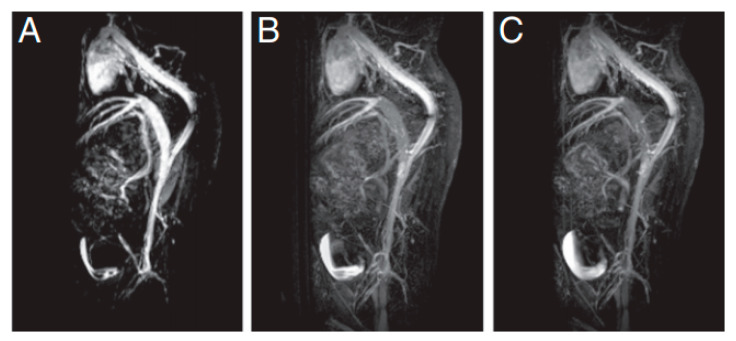
T_1_-weighted MR angiography, at 7 T, of a mouse (**A**) 4 min; (**B**) 12 min; and (**C**) 20 min, after the injection of ZES-SPIONs. Reprinted from H. Wei, O. T. Bruns, M. G. Kaul, E. C. Hansen, M. Barch, A. Wiśniowska, O. Chen, Y. Chen, N. Li, S. Okada, J. M. Cordero, M. Heine, C. T. Farrar, D. M. Montana, G. Adam, H. Ittrich, A. Jasanoff, P. Nielsen and M.G. Bawendi; “Exceedingly small iron oxide nanoparticles as positive MRI contrast agents”; Proceedings of the National Academy of Sciences; 114(9); 2325-2330 (2017). **Copyright (2017)** National Academy of Sciences.

Several authors have also encouraged the use of iron oxide NPs as T_1_ contrast agents, since in clinical practice, typically employed contrast agents are Gd complexes, which, as mentioned, pose health risks [5,10,92,93]. Within this context, Wei et al. [94] investigated zwitterion-coated SPIONs (ZES-SPIONs), possessing inorganic cores with a size of ∼3 nm and an ultrathin hydrophilic shell (∼1 nm). These NPs were shown to present a r_2_/r_1_ ratio equal to 2.0, i.e., a value lower than that associated with other SPION-based positive contrast agents, albeit this was within a factor of 2 to that exhibited by Gd-based chelates. Additionally, in vivo MRI was performed on mice injected with ZES-SPIONs to assess their preclinical potential as positive contrast agents for MRI and MR angiography (Figure 11). Such tests revealed a contrast power that was sufficiently high for use in the considered applications. An efficient renal clearance of the ZES-SPIONs was observed, and by measuring their r_2_/r_1_ ratio again after excretion, the authors verified that the MR contrast power of the NPs was largely unmodified under physiological conditions.

Yin et al. [95] achieved T_1_ contrast in MRI by employing SPIONs with diameters between 11 and 22 nm in an ultra-low field (ULF) MRI system, which applied a ∼0.13 mT magnetic field at room temperature. This approach made it possible to improve the positive contrast created by such NPs, because under these conditions, their relaxation times were similar to the proton Larmor precession period, indicating a great increase in the r_1_ value. Additionally, their r_2_ was lowered, since the magnetic moments present in the SPIONs were not saturated at this field magnitude. As a result, a r_1_ as high as 615 mM^−1^s^−1^ was obtained for Zn_0.3_Fe_2.7_O_4_ NPs coated with silicon dioxide and with a size of 18 nm, being 100 times greater than that of typical commercial Gd-based positive contrast agents under large magnetic fields, i.e., 1.5 and 3.0 T. Furthermore, the authors verified a linear dependence of r_1_ on the imaginary part of the magnetic AC mass susceptibility at 5.56 kHz, i.e., the proton resonance frequency, for all the studied cases. This result was attributed to the NP magnetic fluctuations associated with Brownian motion or Néel relaxation. As a conclusion, various benefits were observed with this approach, namely, adjustable magnetic susceptibility in SPIONs, improved signals, shorter imaging times, as well as the use of biocompatible substances.

**Figure 12:**
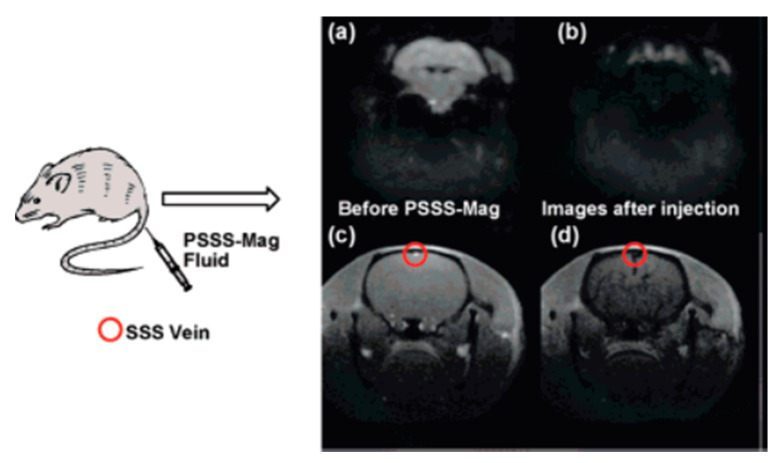
Echo planar image (EPI) of a mouse brain (**a**) before and (**b**) as PSSS-Mag1(Fe/Polysodium-4-styrene sulfonate ratio 1:2) travels across the organ; Fast Low Angle Shot (FLASH) picture of a mouse brain (**c**) before and (**d**) as PSSS-Mag1 travels across the organ. Reprinted with permission from S. A. Corr, S. J. Byrne, R. Tekoriute, C. J. Meledandri, D. F. Brougham, M. Lynch, C. Kerskens, L. O’Dwyer and Y. K. Gun’ko; “Linear Assemblies of Magnetic Nanoparticles as MRI Contrast Agents”; Journal of the American Chemical Society; 130(13); 4214-4215 (2008). Copyright (2008) American Chemical Society.

Also for MRI applications, Corr et al. [45] studied suspensions composed of rectilinear chains of magnetite NPs which were fabricated through a process involving the cross-linking of neighboring nanoparticles with polyelectrolytic complexes. Then, a magnetic field was applied leading, to the reorganization of such nanoarchitectures into collateral arrangements. The relaxivity of the obtained nanostructures was assessed through field-cycling NMR at 37 °C, showing a substantial lowering of the relaxation times for every field used. Additionally, MR pictures of living rats to which such nanostructures were administered were obtained to evaluate the impact that they had upon the brain. Not only was an acceptable biological compatibility for these nanoarchitectures observed, but in vivo MRI images also showed their potential as T_1_-contrast agents, since they obscured brain areas, as shown in Figure 12.

β-glucan particles (GPs) obtained from yeast constitute a category of microcarriers. These are under development; nevertheless, they possess the ability to target cells in the immune system, allowing, for example, the transportation of drugs or imaging agents to those biological entities. However, the encapsulation of these compounds inside porous GPs is difficult. This problem was addressed by Patel et al. [46] through the synthesis of large spin Fe(III) macrocyclic compounds which effectively work as in vivo T_1_ MRI contrast agents. As a result, Fe(III)-based macrocyclic T_1_ MRI contrast agents were easily encapsulated inside the GPs, due to their distinctive coordination chemistry. Furthermore, the GPs labelled with the basic Fe(III) compounds were stable in physiologic environments. Additionally, and in contrast to the free Fe(III) coordination complex, the labelled Fe(III)-GPs not only reduced the T_1_ relaxivity, but also acted as a silenced version of the considered contrast agent.

Recently, Kozlova et al. reported that the control of the contrast of an MRI scan can be achieved by adjusting the nanoparticle concentration in the administrated suspension, or by changing the number of nanoparticles in one carrier. In the same work, it was also reported that the core-shell structure of the nanoparticle influences the contrast efficiency [96]. The authors fabricated polymeric submicron particles with a core-shell structure and verified an improvement of T_1_ and T_2_ gradient contrast when magnetite nanoparticles were incorporated only in the carrier shell. The obtained result was associated with the magnetic interactions that were determined by the package density of the magnetite nanoparticles in the carrier.

## 5. Gd and Mn-based Nanomaterials

Many methods have been developed to incorporate Gd chelates into nanoparticles [15], and lots of studies have focused on the development of silica nanostructures doped with Gadolinium atoms. In this context, Rieter et al. [97] produced stables nanoparticles with r_1_ values higher than those reported by using conventional Gd chelates by using a luminescent core [$\mathrm{Ru}{\left(\mathrm{bpy}\right)}_{3}$]${\mathrm{Cl}}_{3}$ with a silylated Gd complex coating. Another work demonstrated that the relaxometric properties of the complex strictly depend on where the Gd complex in mesoporous silica nanoparticles (MSNs) is placed. The highest r_1_ value of 33.6 ± 1.3 ${\mathrm{mM}}^{-1}s^{-1}$ was found to be related with a specific synthesis method, namely, the long-delay, co-condensation process. The reported relaxometric value was larger than those of Gd-DOTA silica NPs, and much greater than the unbound Gd-DOTA (~20 times) [98]. Biotin was covalently attached to the structures, which led to complexes, after the conformational change, with a large relaxivity, but displaying permutable behavior in the presence of a specific protein target [99]. To integrate Gd-DOTA components, graphene oxide (GO) was also used as a viable platform. A study by Zhang et al. [100] reported that PEG chains were first covalently attached to the GO complex with subsequent surface modification by DOTA, and then Gd(III) ions were implanted. These nanostructures, under the application of a 11.7 T field, showed an r_1_ value of 14.2 ${\mathrm{mM}}^{-1}s^{-1}$. Some other strategies to incorporate Gd into several types of nanoparticles have also been reported, namely by grafting Gd(III) in detonation nanodiamond (DND) [101] and melanin-dots (M-dots) loaded with Gd (II) [102].

**Figure 13:**
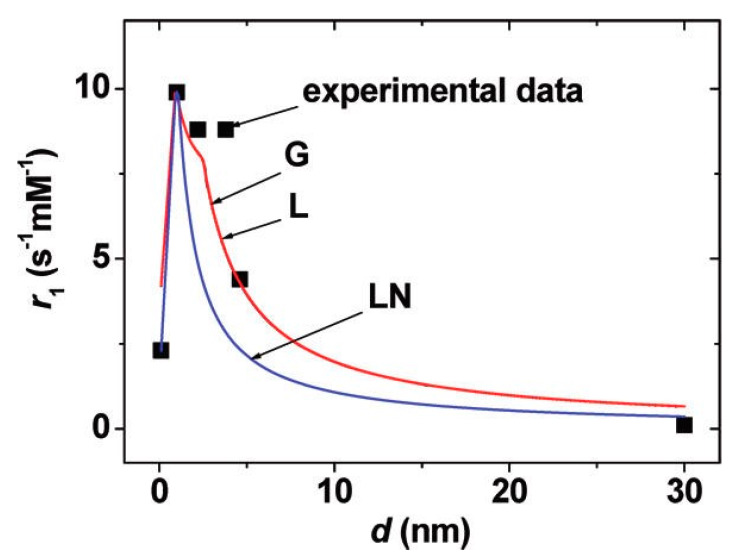
Reproductions of r_1_. The functions are labeled as G (Gaussian), L (Lorentzian), and LN (log-normal). Reprinted with permission from J. Y. Park, M. J. Baek, E. S. Choi, S. Woo, J. H. Kim, T. J. Kim, J. C. Jung, K. S. Chae, Y. Chang and G. H. Lee; “Paramagnetic ultrasmall gadolinium oxide nanoparticles as advanced T_1_ MRI contrast agent: account for large longitudinal relaxivity, optimal particle diameter, and in vivo T_1_ MR images"; ACS nano; 3(11); 3663-3669 (2009). Copyright (2009) American Chemical Society.

Gadolinium oxides are considered the most suitable option for Gd chelates; it has been stated that a decrease in the diameter of the particle will lead to an increase in the relaxivity up to a maximum value. As an example, Park et al. [20] showed that the lower the dimension of the particle, the higher the relaxivity and maximum value reached when the diameter of the structure, d, is between 1–2.5 nm, as presented in Figure 13. Consequently, ultrasmall gadolinium oxide nanoparticles have been used in in vivo studies to image a tumor in a rat’s brain. The enhanced contrast of the T_1_-weighted sequences was considered the be the result of the combination of the intrinsic large surface structure compared to the core-shell, and the presence of Gd(III) ions attached to the surrounding area. Nonetheless, ultrasmall ${\mathrm{Gd}}_{2}O_{3}$ NPs have been found to create aggregates in the cerebrum; as a result, it can be said that the toxicity of the particles is proportional to the maximization of the image potency. Yin et al. [103] developed gadolinium oxide-coated silica nanostructures by exploring different thicknesses of the ${\mathrm{Gd}}_{2}O_{3}$ component. It was demonstrated by varying the thickness of the silica shell over a wide range that with thinner shell values, larger r_1_ values can be achieved. In addition, the nanocomposites showed no significant issues in terms of toxicity. The enhanced signals in in vivo tumor-targeted MRI indicated that ultrasmall gadolinium oxide nanoparticles with a core-shell structure are good candidates as T_1_ contrast enhancing agents for clinical trials.

Dual T_1_- and T_2_-weighted MRI agents were also reported by Zeng et al. [104]. These authors fabricated nontoxic gadolinium hybrid iron oxide (GdIO) nanoarchitectures with hydrodynamic size between 120 and 150 nm. These nanocomposites exhibited both unusual paramagnetic and superparamagnetic behavior and their magnetic moments, measured at 5T in a nonsaturated point of the hysteresis curve, were found to be 33.5 emu/g The GdIO samples exhibited high contrast ability for use as both positive and negative contrast agents, with a Gd-based positive relaxivity value of 70.10 ± 3.65 mM^−1^ s^−1^ and an Fe-based negative relaxivity value of 173.55 ± 6.48 mM^−1^ s^−1^. Through in vivo trials, it was shown that the GdIO nanocomposites were capable of reaching tissues in the cerebrum and further moving toward somatic cells [104].

Bailey et al. [44] reported the fabrication of ${\mathrm{RE}}_{2}O_{3}$-based nanodiscs, exploring diameters in the range of 10–14 nm; RE stands for Gd, dysprosium (Dy) or ytterbium (Yb) passivated with a nontoxic polymer. Here, their appropriateness for use as MRI contrast agents was analyzed. To be able to compare the relaxation times of such nanostructures, either to spherical ones or small DTPA ligand-base chelates, a magnetic field of 1.41 T and a temperature of 37 °C (body temperature) were adopted. The authors also executed $T_{1}$- weighted MR scans of an appropriate model of the body for the ensemble of samples, revealing that ${\mathrm{Gd}}_{2}O_{3}$ nanodiscs were more suitable as contrast agents than the commercially available Gd-DTPA due to their higher relaxivities [105].The efficiency of in vivo targeted imaging should be increased by the higher relaxivity values, and a large amount of proton relaxation would be achieved, without the need for a large amount nanocomposites attached to the surface of the structure to be imaged. Moreover, it was shown that these ${\mathrm{Gd}}_{2}O_{3}$ nanodiscs could serve as $T_{1}$ contrast enhancing agents. The polymer-coated ${\mathrm{Gd}}_{2}O_{3}$ and ${\mathrm{Dy}}_{2}O_{3}$ nanoarchitectures, on a cell line derived from a human cervical cancer (HeLa), showed negligible cytotoxic effects.

**Figure 14:**
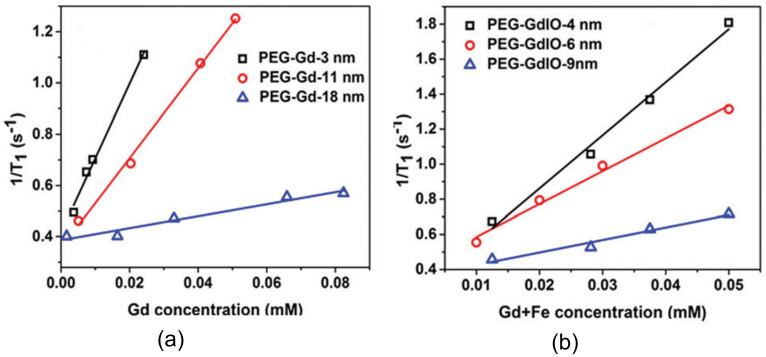
T_1_ relaxation rate versus the accumulation of nanocomposites originating from (left) Gd_2_O_3_ nanodiscs with different sizes and (right) spherical GdIO nanoparticles with a diameter of 6 nm and 9 nm, as well as squared nanostructures with a side of 4 nm. Reproduced with permission from G. Singh, B. H. McDonagh, S. Hak, D. Peddis, S. Bandopadhyay, I. Sandvig, A. Sandvig and W. R. Glomm; “Synthesis of gadolinium oxide nanodisks and gadolinium doped iron oxide nanoparticles for MR contrast agents”; Journal of Materials Chemistry B; 5(3); 418-422 (2017), from The Royal Society of Chemistry.

Singh et al. [23] also reported two different PEG coated structures as viable alternatives to commonly used Gd-based complexes, i.e., gadolinium oxide nanoparticles and Gd-doped iron oxide (GdIO) superparamagnetic nanoarchitectures with different shapes and dimensions. In this report, a 7 T MR scanner was used to measure the relaxivity values of the different nanostructures. It was concluded that the smaller the size of the particle (<5 nm), the higher its relaxivity value (i.e., suitability as $T_{1}$ contrast agents), as presented in Figure 14.

Besides Gd-based nanocomposites, Mn has also been studied as a possible T_1_ contrast agent due to its higher biocompatibility, leading to fewer toxic compounds compared Gd; however, it possesses low intrinsic r_1_ relaxivities. Nevertheless, it has shown promising results as dual modal imaging agent, as presented in Figure 5 [15]. Much effort has been put into increasing biocompatibility and r_1_ by using derived nanoparticulate systems. For instance, polyethylene glycol-coated ${\mathrm{Mn}}_{3}O_{4}$ nanoarchitectures were enclosed in a nontoxic, nanosized porous carbon structure. Then, it the suitability of the nanoarchitecture as a T_1_ contrast enhancing agent was demonstrated. The nontoxicity and ease of dissolution in water of the structure were attributed both to the presence of a carbon structure and the functionalization of the surface, confirming the usefulness of such structures in the interpretation of MRI scans [22].

Neves et al. [47] studied Mn oxide (MnO) NPs (average size of ~20 nm) coated with carboxymethyl-dextran. Despite not having performed an in vivo study, the authors considered such nanostructures to be suitable as T_1_ contrast agents after performing a clinical trial on a 3.0 T MRI scanner, because of their considerable longitudinal relaxivity. Additionally, it was observed that for a small number of particles (less than 25 µg/mL), the NPs were biocompatible in healthy cells; it was also reported that few nanoparticles in solution (5 µg/mL) would result in nonnegligible toxicity effects in the case of HeLa cells.

Manganese ferrite nanoparticles (Fe_3_O_4_@MnIO) have also exhibited larger r_1_ compared to iron oxide NPs [106]. Here, the authors found a longitudinal relaxation value of 33.8 mM^−1^s^−1^ for Fe_3_O_4_@MnIO and a r_2_ of 306.3 mM^−1^s^−1^. The higher r_1_ value was related to the elevated relaxation time of the electronic component and to the presence of a larger number of unpaired electrons due to Fe substitution by Mn ions. Additionally, in vivo results indicated that these nanoparticles may be suitable for in vivo scans, with negligible toxicity at a dosage of 1 mg/kg.

An alternative to the common MRI scan is fluorine (F) magnetic resonance imaging. One of the major drawbacks of common MRI is the presence of noisy signals that arise from the proton nuclei situated in vicinity of the region of interest; in this context, fluorine has been considered a viable alternative, as it possesses adequate magnetic features compared to protons [107]. Despite the advantages of 19-F MRI, in order to reach a satisfactory resolution of the image, a greater amount of contrast enhancing agents has to be employed (from 10 to 50 mM) than with Gd complexes, which are able to generate detectable signals at considerably lower concentrations (0.1 µM) [108]. The low sensitivity of this system arises from two main factors: The low concentration, the long longitudinal relaxation time of 19-F nuclei compared to 1-H, and the fact that a longer T_1_, particularly for diamagnetic complexes, results in an increase in the acquisition time of the scan needed to produce an acceptable image (i.e., with a satisfactory signal-to-noise ratio). To overcome this problem, the longitudinal relaxation rates have to be shortened, a task which is well accomplished by the encapsulation of paramagnetic compounds, such as lanthanides(III) or iron(II), for instance [109].

## 6. Synthetic Antiferromagnetic Nanostructures

More recently, the use of antiferromagnetic nanoarchitectures as T_1_ contrast agents has also been studied by various researchers. For example, Na et al. [21] synthesized antiferromagnetic MnO nanoparticles, presenting dimensions from 7 until 25 nm and possessing a PEG-phospholipid casing. Here, the relaxivity associated with these spherical nanoformations was evaluated in a 3.0 T human clinical scanner. Additionally, their suitability as in vivo MRI contrast agents was assessed in a mouse. It was observed that these NPs were feasible T_1_ contrast agents, demonstrating no significant toxic effects at concentrations under 0.82 mM in eight human cell lines comprising various tissues. Moreover, through their combination with a tumor-specific antibody, the authors were able to selectively enhance the contrast in T_1_-weighted MRI on with breast cancer cells situated in a mouse metastatic brain tumor, into which the nanoparticles with functional groups were intravenously administered.

**Figure 15:**
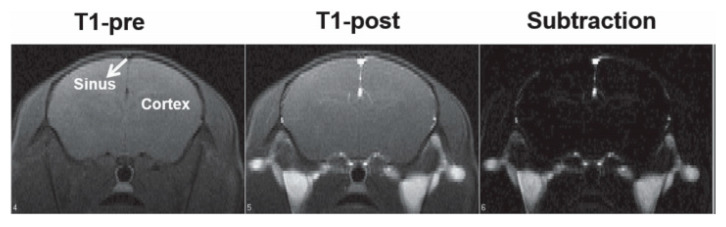
T_1_-weighted MR pictures of mouse brain, prior to (T_1_-pre) and post (T_1_-post) injection of the glutathione-functionalized iron-oxide nanoparticles. Reproduced from C.-L. Liu, Y.-K. Peng, S.-W. Chou, W.-H. Tseng, Y.-J. Tseng, H.-C. Chen, J.-K. Hsiao and P.-T. Chou; “One-Step, Room-Temperature Synthesis of Glutathione-Capped Iron-Oxide Nanoparticles and their Application in In Vivo T1-Weighted Magnetic Resonance Imaging”; Small; 10(19); 3962-3969 (2014). Copyright (2014) WILEY-VCH Verlag GmbH & Co. KGaA, Weinheim.

Liu et al. [110] also fabricated spherical nanostructures exhibiting antiferromagnetic properties. These nanoarchitectures were glutathione-functionalized iron-oxide nanoparticles, produced at room temperature in an aqueous-phase by a facile, extremely efficient, as well as eco-friendly, one-step reduction process, using tetrakis(hydroxymethyl)phosphonium chloride as the reducing agent. After the synthesis procedure, characterization of the nanostructures revealed a diameter of 3.72 ± 0.12 nm, an r_2_ of 8.28 mM^−1^s^−1^, a 2.28 r_2_/r_1_ ratio, reduced magnetization, plus adequate water dispersion. Additionally, through their incubation with HeLa cells, it was observed that they were biocompatible, and that they improved the acquired MR signal intensity in T_1_-weighted sequences, as the iron content inside the considered biological entities increased. Moreover, the fabricated nanoparticles were also injected into mice and rat models to study not only their in vivo circulation and metabolic paths, but also to analyze the contrast they created in MR images under those conditions. The nanostructures escaped the hepatic reticuloendothelial system, and were subsequently expelled from the body via the urinary system, enabling the realization of a renal function assessment. This course led to a long circulation period in the vasculature, providing strong improvement in T_1_-weighted MRI of the vascular resolution of not only the internal carotid artery, but also the superior sagittal sinus, which are locations where the thrombus identification is essential for identifying a stroke (Figure 15). Additionally, various T_1_- and T_2_-weighted MR pictures of a rat’s kidney injected with these nanostructures yielded a detailed visualization of both the cortical-medullary anatomy and the renal physiological functions.

In another study, Peng et al. [46] analyzed a different T_1_ contrast agent type, antiferromagnetic-iron oxide-hydroxide nanocolloids, with a diameter of 2–3 nm. Such nanostructures were produced in the mesopores in worm-like mesoporous silica. Then, their relaxation times were assessed at 40 °C utilizing a 0.47 T Minispec spectrometer. These nanoparticles not only had the lowest r_2_/r_1_ ratio that was known before 2013 for colloidal T_1_ contrast agents based on Fe, but also presented significantly large longitudinal relaxivity. Moreover, the obtained MR pictures showed that these nanocolloids had better performance as T_1_ contrast agents in in vitro (HeLa cells) as well as in in vivo (rat; mouse) MRI, in comparison to ultrasmall iron oxide particles at the nanoscale. Additionally, such nanocolloids displayed great biological compatibility and biodegradability.

**Figure 16:**
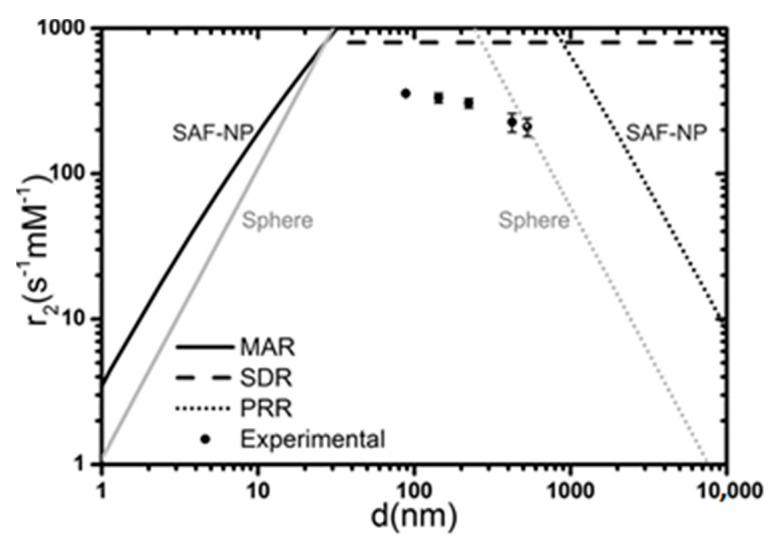
Variation of both the theoretical (dark lines) and the measured (points) r_2_ values associated with [Au (10 nm)/NiFe (10 nm)/Au (2.5 nm)/NiFe (10 nm)/Au (10 nm)] SAF-NPs in terms of diameter. For benchmarking purposes, the theoretical values for NiFe nanoparticles are represented in gray. Reprinted with permission from R. V. >, W. V. Roy, T. Stakenborg, J. Trekker, A. D’Hollander, T. Dresselaers, U. Himmelreich, J. Lammertyn and L. Lagae; “Synthetic Antiferromagnetic Nanoparticles as Potential Contrast Agents in MRI”; ACS Nano; 8(3); 2269-2278 (2014). Copyright (2014) American Chemical Society.

Besides the aforementioned nanostructures, another type of nanoarchitecture investigated for this biomedical application is SAFs. Roosbroeck et al. [61] produced phospholipid-covered, stratified [Au (10 nm)/Ni_80_Fe_20_ (5 nm)/Au (2.5 nm)/Ni_80_Fe_20_ (5 nm)/Au (10 nm)] SAF nanostructures, presenting a disc architecture and exhibiting diameters among 89.8 nm and 523.2 nm, via a colloidal lithography methodology. Their magnetic appraisal indicated a remarkably small remanence, which is necessary to prevent particle agglomeration, plus a large magnetization. These properties suggest that such nanostructures are suitable for use in the biomedical context. Consequently, the authors then assessed the feasibility of these nanoarchitectures as T_2_ contrast agents. This evaluation, as shown in Figure 16, indicated that the nanostructures possessed enhanced relaxivities at 24.85 °C under a 9.4 T magnetic field compared to SPIONs, particularly the smaller ones, with a diameter of 90 nm. Here, the researchers also used these nanoarchitectures in an in vitro MRI investigation, on an ovarian cancer cell line (SKOV3). This analysis showed that a higher T_2_ relaxation existed in the cells labelled with these nanostructures.

## 7. High aspect Ratio Nanowires

**Figure 17:**
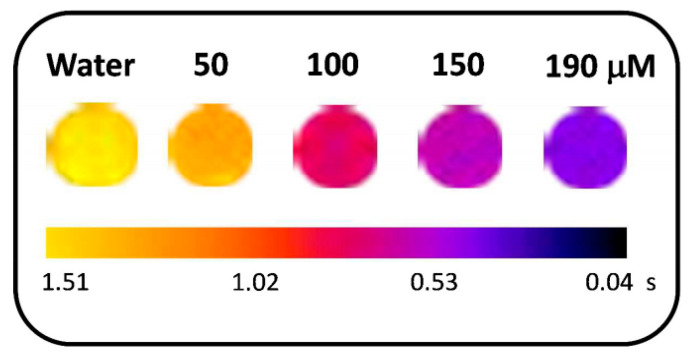
T_2_ map associated with PAA-coated Ni nanowires, obtained at 3 T and at 37 °C. Reproduced with permission from M. Bañobre-López, C. Bran, C. Rodríguez-Abreu, J. Gallo, M. Vázquez and J. Rivas; “A colloidally stable water dispersion of Ni nanowires as an efficient T_2_-MRI contrast agent”; Journal of Materials Chemistry B; 5(18); 3338—3347 (2017), from The Royal Society of Chemistry.

The use of nanowires (NWs) in this particular biomedical application has also been investigated by various authors. For example, Bañobre-López et al. [60] assessed the relaxivity attributes associated with a colloidally stable water dispersion of poly-acrylic acid (PAA)-covered Ni ferromagnetic NWs, characterized by a longitudinal magnetic anisotropy. The fabrication method involved the pulsed electroplating of Ni/Gold (Au) multilayered nanowires within a nanoporous aluminum oxide template at room temperature, followed by template removal and chemical etching of the Au layer in acidic etching involving two stages. Then, the relaxation times associated with the synthesized nanoarchitectures, which had a monodisperse mean diameter and length of ~36 nm and ~600 nm, respectively, were assessed utilizing a relaxometer at a frequency of 60 MHz at 37 °C in two different magnetic fields, specifically 1.41 T and 3.0 T. As a result, it was verified that these nanoarchitectures were efficient T_2_ contrast agents, as is clearly visible in Figure 17. Additionally, the contrast produced by these nanostructures was observed in an MRI scan of a phantom, performed under a 3 T magnetic field.

Shore et al. [39] also studied nanowires for MRI applications. Specifically, Fe and segmented Fe/Au nanowires with various lengths and diameters were fabricated by template-assisted electrodeposition. These nanostructures were covered with certain substances, specifically Dop-PEG and/or SH-PEG-COOH, allowing the linking of biomolecules to the fabricated one-dimensional nanostructures, and permitting the targeting of specific cells. Additionally, the magnetic appraisal of the two nanoarchitectures showed a greater saturation magnetization for the multilayered nanowires. Moreover, since the thickness of their Fe sections was lower than their diameter, such nanoarchitectures were more easily magnetized than pure Fe nanostructures along the orthogonal direction with respect to the long axis of the nanostructure. Furthermore, the relaxivity attributes associated with the fabricated nanoarchitectures were assessed at 25 °C and a 1.5 T magnetic field, and the obtained results were compared to those of Fe as well as Fe-Au nanoparticles. The researchers verified that the 0.7 µm long Fe nanowires with a diameter of 110 nm and encapsulated in Dop-PEG presented the highest potential as T_1_ contrast agents. In contrast, the 1-µm-long Fe-Au nanowires with a diameter equal to 32.8 nm and covered with SH-PEG-COOH plus Dop-PEG showed the best suitability as T_2_ contrast agents, exhibiting a r_2_ similar to that of commercially available Fe oxide nanoparticles. Here, an MR scan was executed of a few samples comprising Fe as well as Fe-Au nanowires at a magnetic field equal to 9.4 to verify the contrast.

Another type of nanowire for biomedical application was investigated by Leung et al. [111]. These nanostructures, made from Mn–Fe, were synthesized through the ligand-induced self-organization of Mn–Fe oxide nanoparticles. Via TEM, it was observed that they possessed a mean diameter equal to 35 nm and, on average, were 1 μm long. The elemental content of the nanowires content was verified by inductively coupled plasma-optical emission spectroscopy (ICP-OES), as well as through energy-dispersive X-ray (EDX) spectroscopy; an Fe percentage of ∼40.65 was indicated. Moreover, their influence on the T_2_ relaxation time was assessed using a 1.5 T MRI system. Here, it was shown that these nanoarchitectures considerably increased the MRI signal decay speed at concentrations of 100 μg/mL or 10 μg/mL, which demonstrated their potential as T_2_ contrast agents. Moreover, the cell labelling efficiency of these nanostructures was assessed by incubating them with a macrophage cell line (RAW264.7). Subsequently, the effective incorporation of Mn–Fe nanowires into the considered biological entities was achieved.

Also considering improvements of the negative contrast in MRI, Martínez-Banderas et al. [112] fabricated distinct, one-dimensional nanostructures composed of an iron core together with an iron oxide shell (Fe-Fe_x_O_y_ core–shell NWs). Their r_1_, r_2_, and r_2_/r_1_ ratio was assessed under a 1.5 T magnetic field; it was observed that they possessed a great potential for this application. Furthermore, the effects of various oxidation levels as well as surface coatings were evaluated under a 7.0 T field. It was shown that their r_2_ could be adjusted by not only the oxide shell thickness, but also by coating agents. Moreover, breast cancer cells (MDA-MB-231) labelled with Fe-Fe_x_O_y_ core–shell NWs, coated with two different compounds, namely bovine serum albumin (BSA) and (3-aminopropyl)triethoxysilane (APTES), were inserted into tissue-mimicking phantoms. Then, T_2_-weighted MR images of these cases were taken; it was observed that the BSA coating improved the dispersion and the cellular internalization compared to APTES, while providing identical cell identification efficiency through MRI. Consequently, this has permitted the use of a lower concentration of nanostructures to efficiently mark the desired cells, thereby lowering the probability of toxicity effects. Using such coating, the authors were able to detect ∼25 cells/μL by employing a NW concentration of 0.8 μg of Fe/mL. Cells labelled with BSA-coated nanostructures were implanted inside a mouse brain and several T_2_-weighted MR images were acquired at a 11.7 T field at various time intervals postimplantation. It was observed that these biological entities could be identified in such organs for at least 40 days after insertion.

The use of iron oxide (Fe_3_O_4_) nanostructures with rod-like morphologies (nanowires with low aspect ratio), diameters of 4–12 nm and lengths ranging from 30 to 70 nm in this specific biomedical application was also examined by Mohapatra et al. [113]. It was verified that nanorods of 70 nm length showed a r_2_ relaxivity of 608 mM^−1^ s^−1^. Additionally, the increase of the size of the nanorods caused a linear increase in their r_2_ relaxivity values, i.e., from 312 to 608 s^−1^mM^−1^. Such trend was attributed to an augmentation associated with both the saturation magnetization and the surface area of the nanostructures. Moreover, in vitro assays considering HeLa cells indicated that those biological entities exhibited normal growth in the presence of Fe_3_O_4_ nanorods, indicating acceptable biocompatibility without toxic effects (approximately 90% the cells remained alive), even postincubation with 1 mg/mL of nanorods.

## 8. Theragnosis Applications

Nanotechnology is a powerful approach for the development of novel nanomaterials that can be used for both the diagnosis and treatment of illnesses, thereby addressing the biggest challenges in cancer therapeutics. A lot of effort has been placed in the study of several types of nanoarchitectures due to their promising applications as “theragnostic” anticancer agents, showing good performance in imaging combined therapy, i.e., hyperthermia, radiotherapy or drug delivery.

Concerning the treatment of tumor diseases, smart drug delivery systems (SDDSs) are considered particularly interesting for theragnostic purposes. Nevertheless, the development of a biocompatible system which is able to combine diagnostic efficiency with the effective distribution of the drug is still the object of investigation. A study by Wang et al. [114] reported the fabrication of nanostructures obtained by a combination of Poly (lactic-co-glycolic) acid (PLGA), indocyanine green (ICG) and iron-based magnetic crystals. These structures have switchable sizes (arising from the thermosensitivity of PLGA polymer), and can be loaded either with chemotherapy or photothermal agents. In vivo trials showed that these nanocapsules are able to be delivered selectively into cancer areas by the application of either a magnetic field or a fluorescent probe, providing nonnegligible therapeutic benefits. The stability of blood circulation is provided by the significantly high initial dimension of these nanocapsules (NCs), while the permeability of the structures within the tumor and the governable release of doxorubicin (DOX) are achieved by the reduction and disintegration of the capsule within the tumor microenvironment. Interestingly, the overproduced reactive oxygen species (ROS) arising from a combination of catalysis reactions and the radiation triggered by the ICG relieved the lack of oxygen for compacted cancers, which is necessary for mitigating the counteraction which occurs due to the insufficient quantity of oxygen during synergetic therapy. Due to the unique properties of such nanostructures, an almost complete elimination of tumors was accomplished. Moreover, a complete scan was achieved by using a dual MRI-fluorescence imaging system. In conclusion, this study has successfully implemented nanocapsules which are highly tumor selective for use either for diagnoses or therapeutic treatment.

**Figure 18:**
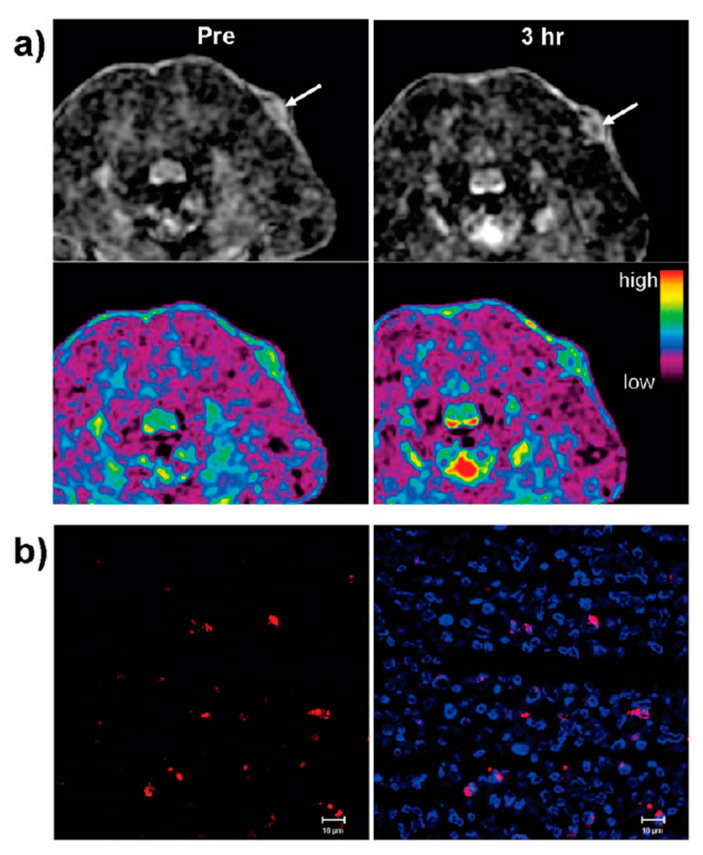
In vivo build-up of Fe_3_O_4_ nanostructures in the tumor area. (**a**) In vivo T_2_-weighted MR scans (upper) and color mapped (lower) images of the region of interest before and 3 h after the introduction of Fe_3_O_4_ nanoparticles into the bloodstream (the arrows illustrate the cancer area). (**b**) Confocal laser scanning microscopic images of detached cancer tissue accumulated the day after injection. Left: Red fluorescence presenting cells in presence of Fe_3_O_4_. Right: Merged image with 4’,6’-diamino-2-fenil-indol (DAPI) stained nuclei (blue) (scale bar 10 μm). Reprinted with permission from J. E. Lee, N. Lee, H. Kim, J. Kim, S. H. Choi, J. H. Kim, T. Kim, I.C. Song, S. P. Park, W. K. Moon and T. Hyeon; “Uniform Mesoporous Dye-Doped Silica Nanoparticles Decorated with Multiple Magnetite Nanocrystals for Simultaneous Enhanced Magnetic Resonance Imaging, Fluorescence Imaging, and Drug Delivery”; Journal of the American Chemical Society; 132(2); 552-557 (2010). Copyright (2010) American Chemical Society.

Until now, among the different architectures, specifically designed magnetite nanostructures have shown the greatest potential for use as both diagnostic and therapeutic agents due to their adaptability, nontoxicity, ease of synthesis and very suitable magnetic properties for the biomedical field, as presented, for instance, in Figure 18 [115]. With the core-shell strategy, several structures, like single- and multi-core shell NPs, have been explored, where magnetite can be located in either the core, in the shell or enclosed in a polymer mold. In all cases, the multimodal features of the nanostructures are the result of a combination of attributes, and make possible the simultaneous use of different techniques in combination, such as contrast enhancing agents in MRI/Positron emission tomography (PET) [116].

A study by Efremova et al. [117] reported the fabrication of octahedral-shaped Fe_3_O_4_ magnetite nanoparticles with diameter of 25 nm grown on 9 nm gold nanospheres. The nanoarchitectures exhibited magnetic features comparable to those of bulk materials. Additionally, it was verified through in vitro and in vivo assays that the nonpure structures, because of their shape anisotropy, presented higher transverse relaxivity for MRI compared to conventional contrast enhancing agents. Furthermore, iron oxide-gold nanocomposites were associated with either two fluorescent pigments or a conjunction of a pigment and a drug, making them able to simultaneously detect the pattern of the carrier and the loaded drug. Furthermore, by using specific microscopy techniques, the authors were able to detect in real time the consignment and leak of the drug in a cancer area. Moreover, by substituting the pigments with particles that bind to specific cells, iron oxide-gold nanoarchitectures appear to be good candidates for theragnostic applications. Gold and SPION-loaded micelles were also used as theragnostic agents to image and treat cerebrum cancers [118]. Other magnetic-gold nanoarchitectures can be found in the literature as alternatives for theragnosis applications [119,120].

Besides the structures reported above, self-assembled peptide hydrogels have recently developed as a novel prototype in biomaterial research. For instance, in a report by Carvalho et al. [121], new dehydrodipeptides consisting of specific amino-acids were synthetized and analyzed. SPIONs were integrated into the hydrogels and the resulting structures were fully characterized in order to understand their new properties. The authors successfully demonstrated that self-build hydrogels maintain their magnetic features when SPIONs are incorporated in the structure, regardless of the presence of a diamagnetic component which arises from the hydrogel grid. Dual T_1_/T_2_ MRI contrast agents were obtained, and it was observed that the SPIONs, with the application of an alternating magnetic field, were able to produce an increase in the local temperature that was sufficient to trigger a phase transition of specific hydrogels; in this context, the leak of SPIONs from the hydrogel compounds upon the application of an alternating magnetic field could a successful step in the development of more powerful theragnostic systems.

## 9. Prospects and Conclusions

Progress in the development of contrast agents for MRI is notoriously slow. Beyond the various contrast agents which have been approved for use in the humans, there are a significant number of new agents in clinical and research development. There is a broad variety of fabrication procedures to obtain paramagnetic and superparamagnetic nanoparticles with control over their composition, size and shape. Furthermore, the ability to integrate additional imaging modes or drugs for treatment, and the use of specific vectors linked to the particle surface, make these agents yet more promising. More recently, other exciting spin configurations have been presented in the literature as potential MRI contrast agents, namely synthetic antiferromagnets (SAFs) and high aspect ratio nanowires (NWs). Biocompatible SAF nanostructures have been reported with coupling between ferromagnetic layers; their relaxivities and saturation magnetization make them promising candidates for this biomedical application. Also, the characteristics of magnetic NWs and segmented NWs have been explored. It was observed that the high surface area of these nanostructures and the high magnetic moment also show interesting potential for contrast enhancement. The development and comprehension of different magnetic effects is of the uttermost importance in the pursuit of novel contrast agents in MRI. On the other hand, systematic in vivo studies are also needed to understand their mechanism of action and accumulation in different organs.

## Figures and Tables

**Table 1:** Commercially-approved contrast agents and some examples that have received approval for clinical trials [3,10,17,18].

Trade Name	Generic Name	Administration Route (Category)	Applications	Approval
Dotarem/ Clariscan	Gadoterate meglumine	Intravenous (ECF)	Multipurpose	1989 EU 2013 US
Prohance	Gadoteridol	Intravenous (ECF)	Multipurpose	1992
Gadovist (EU) Gadavist (US)	Gadobutrol	Intravenous (ECF)	Multipurpose	1998 EU 2011 US
Magnevist ^a^	Gadopentetate dimeglumine	Intravenous (ECF)	Multipurpose	1988
Omniscan ^a^	Gadodiamide	Intravenous (ECF)	Multipurpose	1993
Optimark ^a^	Gadoversetamide	Intravenous (ECF)	Multipurpose	1999
Multihance	Gadobenate dimeglumine	Intravenous (ECF)	Liver, Multipurpose	2004
Gadomer	Gadomer-17	Intravenous (ECF)	Multipurpose	-
Vistarem	Gadomelitol	Intravenous (Blood Pool)	Multipurpose	-
Clariscan	PEG-feron (USPIO)	Intravenous (Blood Pool)	Multipurpose	-
Sinerem/ Combidex	Ferumoxtran-10 (USPIO)	Intravenous (Blood Pool)	Multipurpose	-
Ablavar (Vasovist, Angiomark) ^b^	Gadofosveset trisodium	Intravenous (Blood Pool)	Angiography	2005 EU 2008 US
Primovist (EU) Eovist (US)	Disodium gadoxetic acid	Intravenous (organ-specific)	Liver	2005 EU 2008 US
Teslascan	Mangafodipir trisodium	Intravenous (organ-specific)	Liver, myocardium	1997
Endorem (EU) Feridex (US)	Ferumoxides (SPIO)	Intravenous (organ-specific)	Liver	1996 US
-	Sprodiamde	Intravenous (organ-specific)	myocardium, brain perfusions	-
Sinerem/ Combidex	Ferumoxtran-10 (USPIO)	Intravenous (organ-specific)	metastatic lymph nodes, macrophage imaging	-
Feraheme ^c^	Ferumoxytol (USPIO)	Intravenous (organ-specific)	brain lesions, liver, lymph nodes, blood vessels	2009 US 2013 UE
Lumirem/ GastroMARK	ferumoxsil (MPIO)	Oral	Gastrointestinal tract	1996
Ferriseltz	ferric ammonium citrate	Oral	Gastrointestinal tract	1992
Lumenhance	manganese chloride	Oral	Gastrointestinal tract	1997

^a^ Suspended by the European Medicines Agency in 2017. ^b^ Approved for magnetic resonance angiography (MRA) but has been discontinued. ^c^ Approved only for iron replacement therapy.
